# Influence of the Nose Radius on the Machining Forces Induced during AISI-4140 Hard Turning: A CAD-Based and 3D FEM Approach

**DOI:** 10.3390/mi11090798

**Published:** 2020-08-23

**Authors:** Anastasios Tzotzis, César García-Hernández, José-Luis Huertas-Talón, Panagiotis Kyratsis

**Affiliations:** 1 Department of Design and Manufacturing Engineering, University of Zaragoza, 50018 Zaragoza, Spain; cesarg@unizar.es (C.G.-H.); jhuertas@unizar.es (J.-L.H.-T.); 2 Department of Product and Systems Design Engineering, University of Western Macedonia, 50100 Kila Kozani, Greece; pkyratsis@uowm.gr

**Keywords:** AISI4140 turning, machining forces, tool micro-geometry, nose radius, 3D FEM, DEFORM3D, RSM

## Abstract

The present study investigated the performance of three ceramic inserts in terms of the micro-geometry (nose radius and cutting edge type) with the aid of a 3D finite element (FE) model. A set of nine simulation runs was performed according to three levels of cutting speed and feed rate with respect to a predefined depth of cut and tool nose radius. The yielded results were compared to the experimental values that were acquired at identical cutting conditions as the simulated ones for verification purposes. Consequently, two more sets of nine simulations each were carried out so that a total of 27 turning simulation runs would adduce. The two extra sets corresponded to the same cutting conditions, but to different cutting tools (with varied nose radius). Moreover, a prediction model was established based on statistical methodologies such as the response surface methodology (RSM) and the analysis of variance (ANOVA), further investigating the relationship between the critical parameters (cutting speed, feed rate, and nose radius) and their influence on the generated turning force components. The comparison between the experimental values of the cutting force components and the simulated ones demonstrated an increased correlation that exceeded 89%. Similarly, the values derived from the statistical model were in compliance with the equivalent FE model values due to the verified adequacy.

## 1. Introduction

Turning is one of the most widely used machining operations in industry. In particular, turning of hardened steel is an indispensable part in a modern manufacturing framework. Moreover, AISI-4140 is preferred for the production of many typical mechanical parts such as gears, shafts, and bearings [1]. For these reasons, a number of studies exist in the literature that investigate various aspects that occur during the machining of hardened steel [2,3,4,5,6]. The methods that are usually implemented in such research usually involve experimental work, statistical, and numerical analyses. However, the number of cases that use the finite element method (FEM) during numerical analysis is not high. Furthermore, the majority of FEM-based numerical studies tend to develop 2D models.

Saez-de-Buruaga et al. [7] proposed a methodology that determined the influence of cutting conditions on the developed cutting temperatures that was compared to the 2D simulated results of the tool/chip contact temperatures. Ye et al. [8] studied high speed cuttings of various metallic materials over wide ranges of cutting speeds. The authors used the model for prediction purposes of the critical cutting speed at which the serrated chip flow occurs. Similarly, Shuang et al. [9] performed 2D cutting simulations to better understand the chip formation and material behavior during high speed machining of the Ti6Al4V alloy. Orthogonal cutting simulations provide researchers with important insights into numerous machining aspects such as the estimation of tool wear, machining forces, temperatures, residual stresses, etc. at reasonable computational cost [4,10,11,12]. Despite the fact that 3D simulations require a hefty amount of computational resources compared to 2D, they do not share the restrictions that two-dimensional simulations have [13]. In addition, three-dimensional FEM enabled by high-end computers provide better visualization and understanding of practical cutting operations [14].

With the aid of 3D FEM, complex operations such as drilling and milling can be studied in order to obtain more information on important parameters such as the developed forces, tool wear, and microstructure [15,16,17]. The three-dimensional FEM has also been used during investigations of turning with the same success. An early example is the work of Guo and Liu [18], who developed a general practical explicit 3D finite element analysis (FEA) model for the analytical purposes of AISI-52100 hard turning with the use of polycrystalline cubic boron nitride (PCBN) inserts. Later, Karpat and Ozel [19] developed a 3D FEM-based model to predict forces and temperatures on various uniform and variable edge micro-geometry PCBN turning inserts. Malakizadi et al. [20] presented a FEM-based approach to predict the flank wear for uncoated cemented carbide tools during conventional turning. Similarly, Lotfi et al. [21] worked on the estimation of the tool wear during turning of Inconel 625 with the aid of FEA. The authors used PVD-TiAlN coated carbide and ceramic inserts in order to study the effect of cutting parameters on the tool wear, temperatures and stress distribution. Hu and Huang [22] examined the effects of cutting speed on the turning force, the temperature distribution, and the tool wear during turning of AISI-4130 with ceramic tools based on 3D FEM. Magalhães et al. [23] presented a finite element (FE) model for hard turning with PCBN inserts prepared with multi-chamfered edges and examined the effects on tool wear and residual stresses.

The present paper investigates the effects of the cutting tool’s micro-geometry, specifically the nose radius, chamfer width, and angle on the generated machining force components during AISI-4140 hard turning. The tools under study were ceramic inserts of the CNGA family and the research was carried out according to three levels of cutting parameters (cutting speed, feed rate, and nose radius) at a certain depth of cut. The numerical simulations were performed with the aid of DEFORM™-3D FEA software so that a FE model could be established. Moreover, a comparison between the experimental results found in the literature [24] and the numerical ones was conducted for validation purposes. Finally, after validating the FE model, a statistical model based on the response surface methodology (RSM) was developed.

## 2. Materials and Methods

### 2.1. CAD-Based Application for Designing Turning Inserts

**Figure 1:**
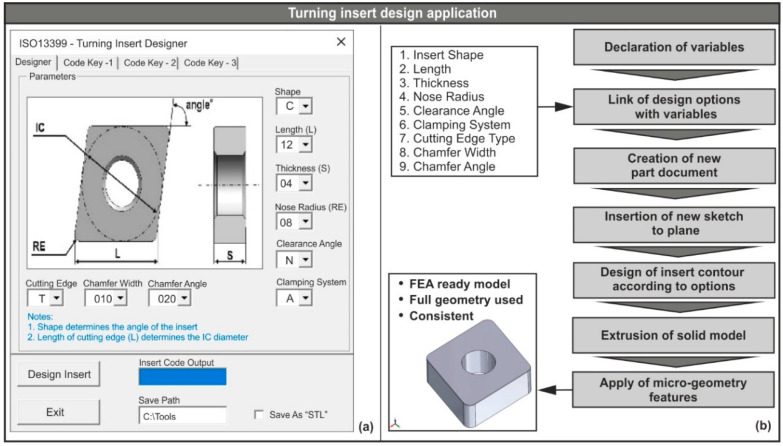
(**a**) The graphical interface of the designer application and (**b**) its workflow.

Nowadays, most tool manufacturers provide CAD models of their products that can be used in 3D simulation runs. However, in most cases, these models are simplified versions and usually miss critical geometric aspects that have important effects on the yielded simulated results. One such geometric parameter is the type of cutting edge. The same conditions also apply to other cutting tools (e.g., drills). In order to overcome this obstacle, a simple yet effective CAD-based application was developed with the aid of the SolidWorks™ application programming interface (API) that accelerated the design process of the insert models used in this study and can be implemented in future projects. Similar work has been done in the past by Vijayaraghavan [25] for drilling tools. The code for this application was developed in Visual Basic for Applications (VBA™) with similar methodologies found in the work of Oancea and Haba [26], and Kyratsis et al. [27]. The graphical interface of the application is illustrated in Figure 1a, whereas Figure 1b contains the workflow. A graphical interface provides users with a simple way to operate the application; in the present case, it was used to select the design parameters according to ISO-13399 standards. Some design parameters are the shape of the insert, the internal circle’s diameter, the cutting edge length, the angle of the corners, the nose radius, the insert’s thickness, and the type of the cutting edge along with the chamfer width and chamfer angle. With the selection of the required parameters, the user can click on the “Design Insert” command button so that the automated design process may begin. According to Figure 1b, the design procedure begins with the declaration of the variables that correspond to each of the geometric characteristics of the insert. Consequently, these parameters are linked with the appropriate variables and a new part of the document is created. Later, a fresh sketch is inserted on the preselected plane and the design of the insert contour takes place based on the parameters seen in Figure 1a (inscribed circle, nose radius, cutting edge length, thickness, corner angle, chamfer width, and chamfer angle). The plane was selected in this way, so that the coordinate system of the model matched the coordinate system of the FEA software. Finally, a solid model of the insert is created with the aid of the “FeatureExtrusion2” method, which can then be saved in both native and “STL” file format. Prior to finalizing the model, the micro-geometry is applied according to the type of cutting edge, the chamfer width, and angle that are selected. In order to apply the aforementioned features to the model, the topology selection routine of the program was executed. With this routine, it is possible to automatically find and select the outer edges of the insert, which can be achieved by traversing all the available edges of the solid model that are then stored to a matrix. Each one of the edges receives a unique name so that they can be called and used at any time. Additionally, the traversal of the edges is achieved with the implementation of a “For” loop. Moreover, the API methods that are responsible for the extraction of the edges and the insertion of the chamfer feature are the “GetEdges” and “InsertFeatureChamfer” accordingly.

The generated solid model is a fully defined, consistent model with the full geometry of the equivalent physical model that can be used in FEA. Even though the tool during machining simulations is defined as rigid and thus its properties are not as critical as the workpiece’s properties [28], the full geometry of the tool, on the other hand, is crucial. Therefore, such models provide increased accuracy during the analysis and the chance of acquiring non-realistic results is minimal.

### 2.2. CAD-Based Layout of the Turning Process

In order to build a simplified analysis domain of the turning process, a CAD-based setup was prepared with the aid of Dassault Systemes (Vélizy-Villacoublay, France) SolidWorks™ 2018. All conditions and geometric characteristics that may affect the cutting process were taken into account. The tool assembly used in this study consisted of a tool-holder and three versions of a conventional rhombic-shaped turning insert. The ISO designation number of the tool-holder was PCBNR2525M12 and the equivalent number for the three inserts was CNGA120404, CNGA120408, and CNGA120412, respectively. The workpiece model was designed as a cylindrical bar with a diameter of 72 mm. Additionally, the applied material for the workpiece was AISI-4140 steel, whereas the insert was ceramic.

**Figure 2:**
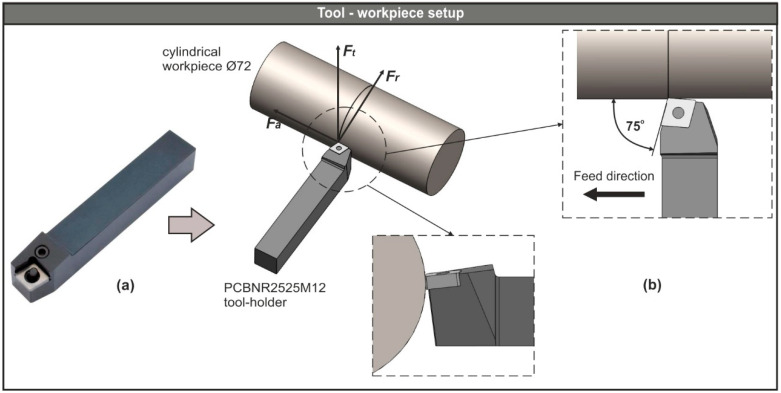
(**a**) The physical model of the PCBNR2525M12 tool-holder and (**b**) the CAD-based tool-workpiece setup.

The physical model of the tool-holder is depicted in Figure 2a, whereas the CAD-based turning process setup is presented in Figure 2b, along with the generated machining forces: *F_t_* is the tangential force, *F_r_* is the radial force, and *F_a_* represents the feed force. Moreover, Figure 2b includes two schematics that focus on the angles related to the cutting process and the feed direction. These angles are inherited from the tool-holder and the turning insert geometry. Thus, the lead angle was 75° and both the rake and inclination angle were negative with a value of −6°.

**Figure 3:**
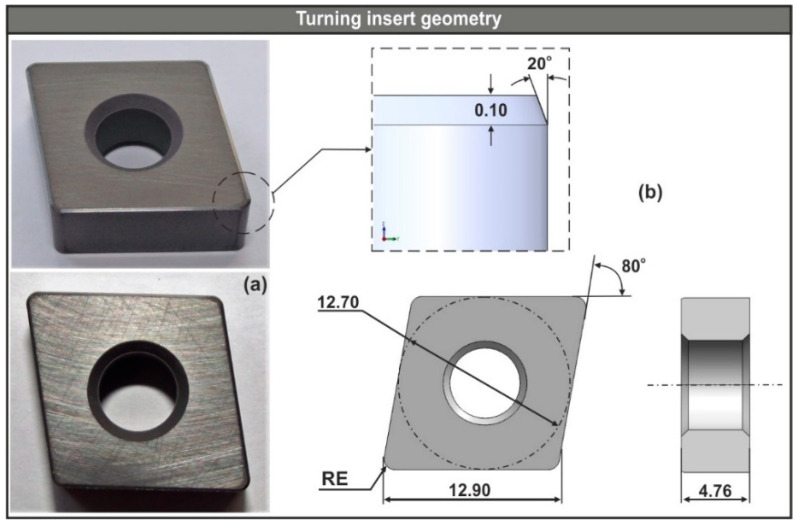
(**a**) The physical model of the CNGA120408T01020 ceramic and (**b**) the analogous CAD model with detailed geometry.

Figure 3a illustrates the physical model of the CNGA120408T01020 (nose radius *re* = 0.80) uncoated ceramic insert, whereas Figure 3b depicts all critical geometric characteristics of the CNGA1204xx turning inserts. The CNGA-ceramic family were negative, 80° rhombic inserts used for machining cast iron and hardened steel.

In the present study, twenty-seven simulation runs were carried out based on the unique combinations of the three levels of cutting conditions: cutting speed (80 m/min, 115 m/min, 150 m/min), feed rate (0.08 mm/rev, 0.11 mm/rev, 0.14 mm/rev), and nose radius (0.40 mm, 0.80 mm, 1.20 mm). The levels of the aforementioned cutting parameters and their values are summarized in Table 1. In addition, the depth of cut was maintained at 0.30 mm for all tests.

### 2.3. Pre-Processing of the 3D FE Turning Model

For the 3D simulation tests, a commercially available FEA software was used, namely SFTC (Columbus, Ohio USA) DEFORM™-3D ver. 12. A desktop PC with six-core CPU 3.60 GHz, 16 GB RAM, and SSD technology hard drive was utilized to carry out the simulations. The completion time for the simulation tests with a feed value of 0.14 mm/rev, 0.11 mm/rev, and 0.08 mm/rev was approximately 6, 10, and 22 h, respectively. The reason for the varied completion time is that the mesh size of the workpiece depends on the selected feed value.

**Figure 4:**
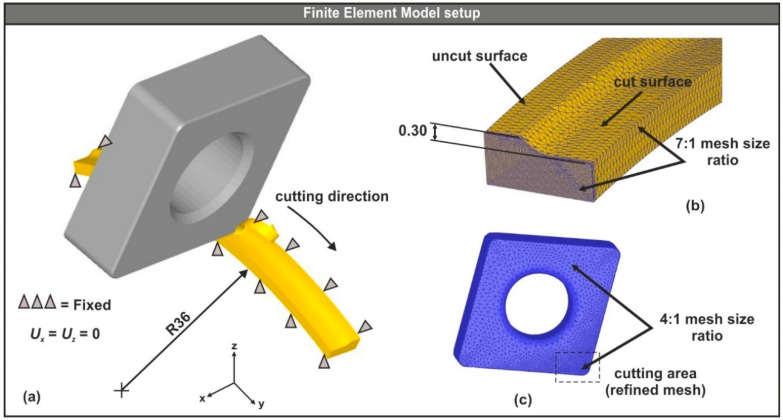
(**a**) The FE model setup, (**b**) the analysis domain, and (**c**) the meshed tool.

After establishing the CAD-based setup of the turning operation, the next step was the preparation of a simplified analysis domain that led to reasonable completion times for the simulation tests. The key points of the simplified setup were the use of the insert model instead of the whole tool assembly and the conversion of the full cylindrical workpiece model to a smaller part, according to the cutting path (circular arc with a diameter of 72 mm and an angle of 45°—see Figure 4a). To further improve the simulation times, the workpiece was designed partially cut based on the depth of cut (Figure 4b).

#### 2.3.1. Configuration of the Insert-Workpiece Interface

The models of the three turning inserts were designed according to the ISO-13399 norms (see Figure 3b), whereas the simplified version of the workpiece was designed with respect to the depth of cut, the nose radius of the tool and the diameter of the steel bar. Moreover, the workpiece was modeled as deformable (plastic) with a mesh that varied between 100,000 and 150,000 elements depending on the size of the minimum element, which was fixed to 25% of the feed value for all tests [29]. Since the area of interest is at the uncut surface of the workpiece, where contact between the insert and the workpiece exists, a finer mesh was applied with a 7:1 ratio (see Figure 4b). In contrast, the insert was modeled as rigid and meshed with the maximum allowed number of tetrahedral elements, which was approximately 50,000 for this case. Furthermore, in order to increase the fidelity of the area near the cutting edge that is in contact with the uncut surface of the workpiece, the related mesh was locally refined with a size ratio of 4:1 (see Figure 4c).

Mesh definition is the parameter that most affects the simulation performance. In order to maintain both the geometry of the chip during forming and the required state variables in the target areas, an adaptive remeshing technique was implemented. The goal of this method is to adoptively refine the mesh of the workpiece so that after a specified number of time steps, the minimum required number of mesh elements is preserved. Hence, the quality of the simulation results were kept to a satisfactory level and at the same time, the simulation runs required a reasonable amount of time to complete.

Finally, the deformation and thermal boundary conditions were set. Specifically, the workpiece was fixed according to Figure 4a, so that the velocity of the nodes in both the X and Z axes was set to zero. In contrast, the insert model was allowed to follow the cutting path, as shown in Figure 4a. The heat exchange between the surface elements of the workpiece and the tool for both convection and conduction were also defined. The value for the heat transfer coefficient via convection that was used in this study was 0.02 N/(s × mm × °C) for dry cutting and via conduction, it was 45 N/(s × mm × °C). These values are the ones provided by default in the DEFORM™-3D software [13].

#### 2.3.2. Modeling of the Insert-Workpiece Materials

The Johnson–Cook material model was used in this study to represent the mechanical behavior of the workpiece. This model is widely accepted by researchers, especially when high strain, strain rate, and temperatures are present during the process. According to Melkote et al. [30], this model has a simple form and is easy to implement and calibrate. The following analytical expression (Equation (1)) constitutes the strain hardening properties of the material, the strain rate sensitivity, and the thermal softening properties accordingly.

(1) $$\sigma =\left(A+B\varepsilon^{n}\right)\left(1+C\ln\frac{\dot{\varepsilon }}{{\dot{\varepsilon }}_{0}}\right)\left[1-{\left(\frac{T-T_{0}}{T_{m}-T_{0}}\right)}^{m}\right]$$

In the previous formula, *σ* is the equivalent stress; *A* is the initial yield stress; *B* is the strain hardening modulus; *C* denotes the strain rate dependence coefficient; *ε* represents the plastic strain; *n* is the strain hardening exponent; *m* is the thermal softening coefficient; and $\dot{\varepsilon }$ is the plastic strain rate, whereas ${\dot{\varepsilon }}_{0}$ is the reference plastic strain rate; finally *T*, *T*_0_, and *T_m_* stand for the reference temperature, the ambient temperature, and the melting temperature of the workpiece material, respectively. To adapt the model for the present case, the constants that are available in Table 2 for the AISI-4140 flow stress were used. All the important properties as well as the model constants of the steel material are available in the software’s library.

Table 3 contains both the mechanical and the thermal properties that were used in the present numerical study. In particular, the elastic modulus, the thermal expansion, the thermal conductivity, and the heat capacity of AISI-4140 were expressed as a function of temperature *f* (Temp) due to the fact that they are temperature dependent. In addition, a reference strain rate of 1/s was used.

In order to approximate the fracture of the material that occurs due to the material separation under the action of stress, the normalized Cockroft–Latham damage model was employed.

Equation (2) represents the modified criterion developed by Cockroft and Latham [32]. In this formula, the maximum principal stress is normalized by the effective stress.

(2) $$D_{c}=\int_{0}^{\varepsilon_{f}}\frac{\sigma_{\max}}{\bar{\sigma }}d\varepsilon_{pl}$$

In the integral, Dc is the material constant in the fracture criterion; *σ_max_* is the maximum tensile principal stress; $\bar{\sigma }$ denotes the effective stress; *ε_f_* represents the limit fracture strain; and finally, *ε_pl_* stands for the plastic strain. This criterion is widely accepted and was implemented in early FE studies such as in the formability of solid cylindrical and ring test specimens by Kobayashi and Lee [33] as well as in the determination of workability in bar extrusion and drawing by Oh et al. [34]. Later, Oyane et al. [35] attempted to predict the fracture strain in actual metal working processes using the basic criterion.

Modeling of the friction situation between two bodies is a very complicated problem, especially when extreme contact pressures develop such as in the case of machining. In order to approximate the phenomena that occur during the contact between the insert and the workpiece, Coulomb’s law was utilized. In the work of Zorev [36], the contact area between the cutting tool and the machined workpiece was divided into sticking and sliding zones. With this in mind, Equation (3) [37] can be used to estimate the developed frictional stresses according to Coulomb’s law.

(3) $$\tau_{f}=\mu \sigma_{n}$$

where *τ_f_* is the frictional shear stress; *μ* denotes the shear friction coefficient; and *σ_n_* represents the tool-chip interface stress. Previous studies [38,39] suggest a value of friction coefficient between 0.5 and 0.6 when studying the machining of AISI-4140 steel at cutting speeds and feed rates similar to the ones used in the present work. Considering the conditions utilized in this research, the shear friction coefficient for the numerical model was set to 0.577 [40].

## 3. Results and Discussion

### 3.1. Assessment of the Cutting Force Components Using FEM

**Figure 5:**
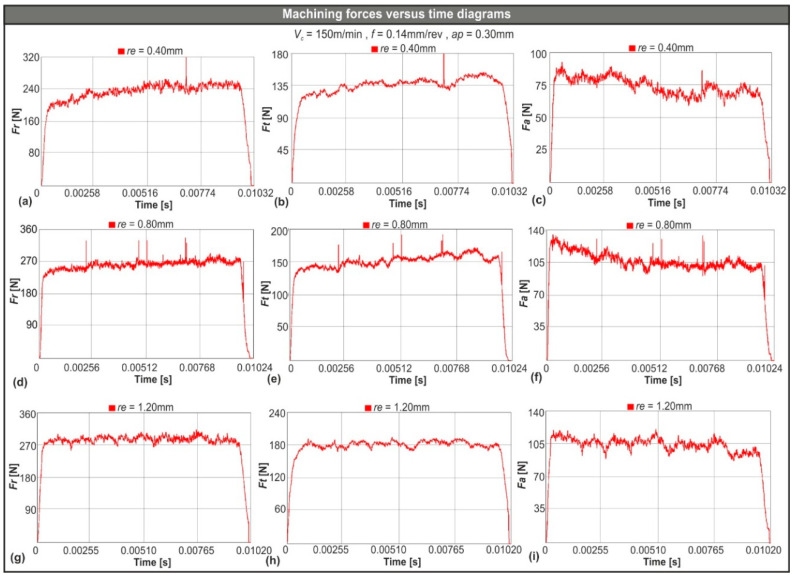
(**a**–**c**) Sample machining forces versus time diagrams for 0.40 mm nose radius, (**d**–**f**) 0.80 mm, and (**g**–**i**) 1.20 mm.

A sample of the generated cutting force components versus time diagrams based on the 3D numerical model are depicted in Figure 5. In particular, Figure 5a–c illustrate the aforementioned diagrams for the radial force (*F_r_*), the tangential force (*F_t_*), and the feed force (*F_a_*) that were generated during AISI-4140 hard turning with the CNGA120404 (*re* = 0.40 mm) insert. Similarly, Figure 5d–f corresponded to the CNGA120408 (*re* = 0.80 mm) insert and consequently Figure 5g–i relate to the CNGA120412 (*re* = 1.20 mm) insert. The following cutting conditions apply for all the previously mentioned sets: *V_c_* = 150 m/min, *f* = 0.14 mm/rev, and *ap* = 0.30 mm. All force versus time diagrams are divided into two phases: the entry phase where force increases quickly as soon as the tool touches the uncut surface of the material and the following steady state phase where force maintains a steady mean value. Finally, when the tool finishes its pass on the workpiece and material removal ends, the force value rapidly decreases until it reaches zero.

Every few time steps, a remeshing procedure occurs in order to keep the necessary minimum amount of elements. Due to this phenomenon, force spikes might appear during the simulation process. Even though the effect of these spikes to the mean value of force is minimal, the default first order exponential smoothing of DEFORM™-3D was enabled to eliminate any unrealistic values.

For the numerical model calibration, a set of experimental values (see Table 4) were used for the exact machining conditions and tools. According to Table 4, the level of agreement between the experimental and the numerical results for *F_main_* is high. Furthermore, based on the findings of Aouici et al. [24] the experimental analysis of the machining components for the next indicative conditions: *V_c_* = 115 m/min, *f* = 0.11 mm/rev, and *ap* = 0.30 mm showed an increased correlation with the equivalent simulated results that were derived from the present study. That is, *F_r_* = 202.3 N, *F_t_* = 146.0 N, and *F_a_* = 86.6 N for the experiments and *F_r_* = 214.4 N, *F_t_* = 137.5 N, and *F_a_* = 77.4 N for the simulations, leading to an estimated relative error of 6.0%, −5.8%, and −10.6%, respectively.

**Figure 6:**
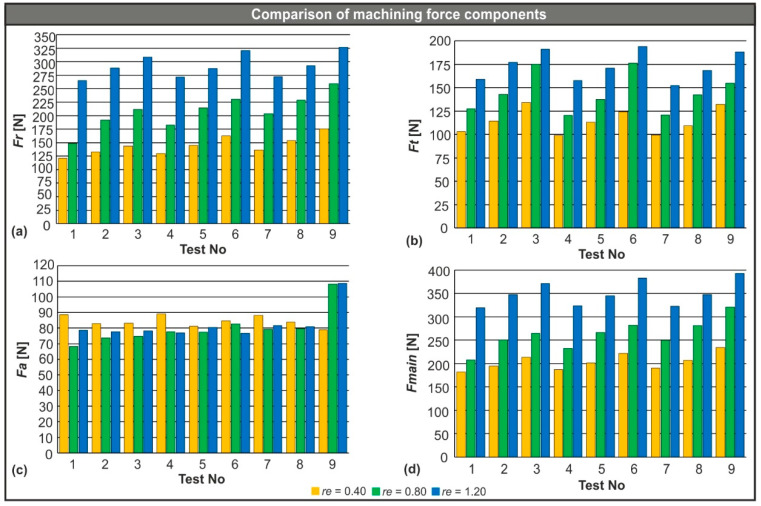
(**a**) Comparison of the radial force, (**b**) the tangential force, (**c**) the feed force, and (**d**) the resultant machining force based on the different nose radii.

With the calibration of the model, two new sets of 3D simulations were performed. Each set consisted of nine simulation tests that were carried out under identical conditions and in the same order as the calibration set. In addition, different tools were used with a nose radius of 0.40 mm and 1.20 mm, respectively (see Table 1). The yielded results were used to plot the charts that are presented in Figure 6. With the plotted results, it is possible to compare the mean values of the generated machining force components for each tool graphically. In particular, Figure 6 contains the results for the radial force (Figure 6a), the tangential force (Figure 6b), the feed force (Figure 6c), and the resultant of the three individual forces (Figure 6d).

Observations of the results from Table 4 and Figure 6 led to the following conclusions:

- The radial force is the component that contributes to the resultant machining force the most. In test number nine, for example, this contribution was approximately 56.6%, 65.3%, and 69.2% for each value of nose radius of 0.40 mm, 0.80 mm, and 1.20 mm, respectively. The same trend was observed in the rest of the tests.
- Any increase in feed rate affects all forces except the feed force. Even though the amount of change is not significant, it cannot be considered negligible either. Specifically, an increase in the feed rate from 0.08 mm/rev to 0.11 mm/rev increased the resultant machining force by about 7.6%, 16.0%, and 7.7% for each nose radius (0.40 mm, 0.80 mm, and 1.20 mm, respectively). Similarly, when the feed rate changed from 0.11 mm/rev to 0.14 mm/rev, the feed rate rose by approximately 10.9%, 13.7%, and 10.4% for the same nose radii, respectively.
- In contrast, the nose radius of the inserts had a notable impact on the generated cutting forces. The main machining force increased by 28% on average when the nose radius of the tool changed from 0.40 mm to 0.80 mm. Furthermore, the tool with the 1.20 mm nose radius produced even higher forces. The change from the 0.80 mm nose radius to the 1.20 mm increased F_main_ by 35% on average.
- Finally, any change in cutting speed had a limited effect on the turning forces. A slight decrease in cutting forces was noted as lower cutting speeds were applied. In particular, by lowering cutting speed from 150 m/min to 115 m/min, the decrease was estimated as approximately 4.5% and for the equivalent shift from 115 m/min to 80 m/min, the reduction was found to be close to 3.4%.

The conditions that were applied in the present research were successfully implemented in previous experimental studies [2,6,41,42,43] for hard turning of similar materials. Davim and Figueira [6] and Quiza et al. [43] used ceramic inserts with designation numbers CNMA120408T01020 and CNGA120408T0120 correspondingly during turning of AISI-D2 at a 0.2 mm depth of cut. Aouici et al. [41,42] reported results of similar magnitude as the present work during turning of both AISI-D3 and AISI-H11 steel with the ceramic SNGA120408T01020 and cubic boron nitride (CBN) SNGA120408S01020 inserts at depths of cut between 0.15 and 0.45 mm, respectively. Furthermore, the authors stated that the radial force was the governing component. In addition, the effects of feed rate and cutting speed were also discussed in the work of Aouici et al. for AISI-4140 [24], clearly verifying the aforementioned observations of the present research.

### 3.2. Modelling of the Resultant Cutting Force Using RSM

In addition to the 3D FE model, a statistically based model was developed with the aid of the response surface methodology (RSM). The purpose of this model was to reduce the number of simulation tests required to predict the cutting forces induced during machining of AISI-4140 under different conditions. The RSM is a well-established statistical tool that formulates a defined relation between two groups of data; one contains dependent variables and the other independent variables. This methodology was embraced by many researchers for prediction and optimization purposes during studies related to typical machining processes [2,3,15,44,45,46] due to the fact that it is versatile and can generate both linear and quadratic models. The three levels of the cutting parameters used (see Table 1) and the number of the numerical tests led to a full factorial design with three factors and a total of twenty-seven experiments. The generated regression model is a second order polynomial that is described by Equation (4). This polynomial includes linear, quadratic, and cross-product terms because the relationship between the input variables and the response is non-linear. In Equation (4), *Y* is the response of the model (resultant machining force), *a*_0_ denotes the fixed term, *X_i_* are the input variables (cutting speed, feed rate and nose radius), and *b_i_*, *b_ij_*, *b_ii_* refer to the vectors that contain the regression coefficients (linear, quadratic, and cross-product, respectively).

(4) $$Y=a_{0}+\sum_{i=1}^{n}b_{i}X_{i}+\sum_{i,j}^{n}b_{ij}X_{i}X_{j}+\sum_{i=1}^{n}b_{ii}X_{i}^{2}$$

Equation (5) presents the complete statistical model for the resultant machining force based on the aforementioned formula and the data of the verified FE model (see Table 4).

(5) $$F_{main}=158.1-0.109V-822f+48.8re+0.00046V^{2}+4504f^{2}+56.6re^{2}+3.03Vf-0.093Vre+498fre$$

In Equation (5), *F_main_* is the resultant machining force in N; *V* is the cutting speed in m/min; *f* is the feed rate in mm/rev; and *re* represents the insert’s nose radius in mm.

Table 5 presents the complete design of experiments that includes all the possible combinations of cutting conditions along with the predicted values of the resultant machining force. The predicted values were derived from both the numerical and the statistical model. Additionally, the comparison between the aforementioned values indicated a strong correlation. The lowest absolute percent error was found to be approximately 6.5% for test number 10, whereas the mean absolute percentage error (MAPE) was close to 2%, which proved the increased agreement between the two models.

### 3.3. Validation of the RSM Based Model

**Figure 7:**
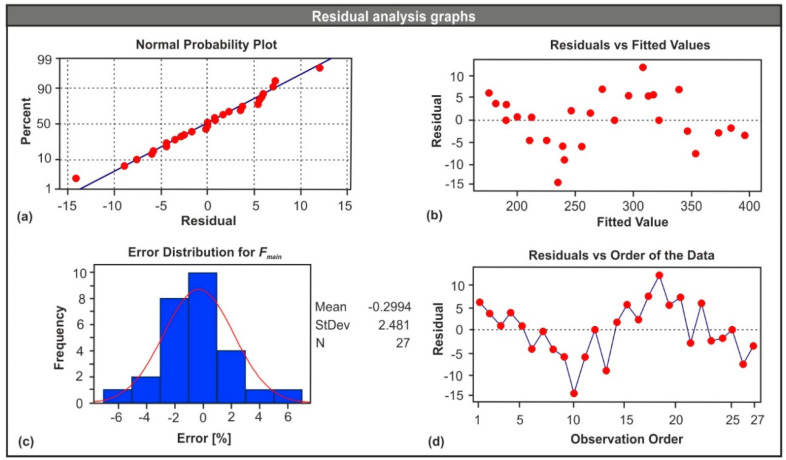
Residual analysis graphs: (**a**) probability plot, (**b**) residuals versus fitted values, (**c**) error histogram, and (**d**) residuals versus order.

The analysis of variance (ANOVA) was employed to validate the model with a standard significance level equal to 0.05. The power of the test was sufficient since the analysis yielded a successful fit of the model with an adjusted R-squared of 98.80% and the obtained ANOVA results are presented in Table 6. The contribution of the factors to the model was highlighted with the aid of the *p*-value. Hence, the constant, the *re*^2^ term and the *f* × *re* term contributed the most, indicating the strong influence of the corner radius to the generated forces. In addition, the total sum of squares was used to express the total variation of the response, which can be visualized with the aid of the data points dispersion graph (see Figure 7a). Last but not least, the *p*-value of the regression model suggests that the probability of acquiring extreme results is thin.

Figure 7 contains the graphical results of the ANOVA that are crucial when checking the validity of the regression model. Specifically, Figure 7a illustrates the normal probability plot, which indicates any departures of the data points from the fit line. In this case, there were no such points. Figure 7b depicts the residuals versus the fitted values plot, which highlights the way the data points are scattered across the reference line. In the present case, it is clear that they were almost evenly scattered on both sides of the line. Figure 7c shows the error percentage histogram along with the fit line in order to check the normality in the error distribution. It is clear that error uniformity was present in the model. Furthermore, the overall normality can be checked by Figure 7d, which illustrates the residuals versus the order of the data. By observing the points in Figure 7d, it can be concluded that no systematic faults were present in the model, since no specific trends or patterns were formed.

**Figure 8:**
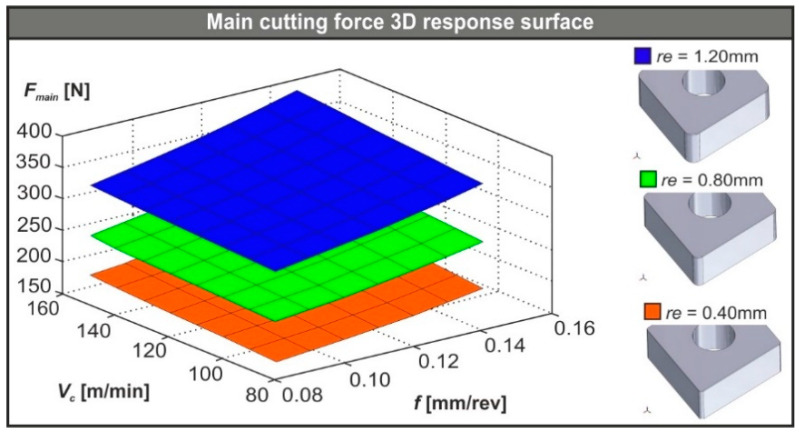
3D plots of the *F_main_* for each nose radius.

Finally, the 3D response surface plots were prepared in order to visualize the performance of the developed regression model according to the data that are available in Figure 6 and Table 5. The performance with respect to the cutting speed, the feed rate, and the tool nose radius are illustrated in Figure 8. Moreover, the 3D plots graphically represent the polynomial solutions of the model according to the range of the cutting conditions found in the present paper. Finally, via the 3D plots it could be observed that:

- The nose radius had a strong impact on the resultant turning force. In fact, an increase from 0.40 mm to 1.20 mm almost doubled the resultant force regardless of the conditions.
- Any increase in feed rate acts as increasing the main cutting force, but at a much lower grade compared to the effect of the nose radius.
- Finally, any change in the cutting speed did not seem to have a significant influence on the main cutting force.

To further examine the validity of the statistical model, six extra simulation tests were carried out with different cutting conditions than the ones used for the establishment of the FE model. However, the parameters were chosen from within the range of the already employed data. Table 7 contains the results for the following conditions at a depth of cut equal to 0.30 mm: I (*V_c_* = 100 m/min, *f* = 0.10 mm/rev, and *re* = 0.40 mm), II (*V_c_* = 120 m/min, *f* = 0.10 mm/rev, and *re* = 0.80 mm), III (*V_c_* = 140 m/min, *f* = 0.10 mm/rev, and *re* = 1.20 mm), IV (*V_c_* = 100 m/min, *f* = 0.12 mm/rev, and *re* = 0.40 mm), V (*V_c_* = 120 m/min, *f* = 0.12 mm/rev, and *re* = 0.80 mm), and VI (*V_c_* = 140 m/min, *f* = 0.12 mm/rev, and *re* = 1.20 mm). The lowest level of agreement (relative error of 10.8%) between the predicted value of *F_main_* and the simulated one was found in test number 5, a fact that indicates that the model provides safe predictions.

## 4. Conclusions

The present paper presented a 3D FE model for the hard turning of AISI-4140 as well as a prediction model for the resultant machining force, based on statistical methods. A set of experimental results that were available in the literature was used to verify the FE model, and consequently, a complete design of experiments was prepared according to three levels of cutting speed, feed rate, and tool nose radius. Further validation of the statistical model was made to ensure that the model could safely predict the resultant cutting force within the range of conditions found in the present study. Additionally, the influence of the nose radius on the produced cutting forces was investigated and graphically presented. Finally, the following conclusions can be drawn:

- *F_r_* is the governing force during hard turning of AISI-4140, which in most cases represents two-thirds of the produced resultant machining force.
- When feed rate changed from 0.08 mm/rev to 0.11 mm/rev *F_main_* gained an average increase of about 10.4%. Similarly, a shift from 0.11 mm/rev to 0.14 mm/rev increased *F_main_* by approximately 11.7%, regardless of the nose radius value.
- The nose radius of the cutting edge affects the generated cutting forces substantially. It was highlighted that a higher value of nose radius leads to higher values of cutting forces, and depending on the applied cutting conditions, the increase percentage exceeded 30% in most cases.
- Finally, changing the cutting speed did not seem to influence the main cutting force notably.

## Figures and Tables

**Table 1:** Process conditions of the turning finite element (FE) model.

Level	*V_c_* (m/min)	*f* (mm/rev)	*re* (mm)
I	80	0.08	0.40
II	115	0.11	0.80
III	150	0.14	1.20

**Table 2:** The Johnson–Cook model constants for AISI-4140 steel [13].

*A* (MPa)	*B* (MPa)	*C*	*n*	*m*	*T*_0_ (°C)	*T_m_* (°C)
106	1167	0.0352	0.1424	0.763	20	1547

**Table 3:** Thermo-mechanical properties for the ceramic insert [31] and steel workpiece [13].

**Mechanical Properties**	**AISI-4140**	**Ceramic**
Young’s Modulus (GPa)	212 @ 20 °C	415
	192 @ 300 °C	
	164 @ 600 °C	
Density (kg/m^3^)	7850	3500
Poisson’s ratio	0.30	0.22
Hardness (HRC)	60	−
**Thermal Properties**	**AISI-4140**	**Ceramic**
Heat capacity (J/kgK)	362 @ 20 °C	334
	446 @ 300 °C	
	610 @ 600 °C	
Thermal expansion (μm/mK)	11.9 @ 20 °C	8.4
	13.6 @ 300 °C	
	14.9 @ 600 °C	
Thermal conductivity (W/mK)	41.7 @ 20 °C	7.5
	41.4 @ 300 °C	
	34.1 @ 600 °C	

**Table 4:** Main machining force comparison between the experimental and numerical values.

		Cutting Parameters			*F_main_* (N)		
Std Order	*V_c_* (m/min)	*f* (mm/rev)	*ap* (mm)	*re* (mm)	Experiments	FE Model	Relative Error (%)
1	80	0.08	0.30	0.80	189.8	207.7	9.4
2	80	0.11	0.30	0.80	244.6	250.2	2.3
3	80	0.14	0.30	0.80	282.3	264.5	−6.3
4	115	0.08	0.30	0.80	225.7	232.1	2.8
5	115	0.11	0.30	0.80	264.1	266.2	0.8
6	115	0.14	0.30	0.80	300.9	281.5	−6.4
7	150	0.08	0.30	0.80	238.0	249.6	4.9
8	150	0.11	0.30	0.80	267.7	281.2	5.1
9	150	0.14	0.30	0.80	316.9	320.7	1.2

**Table 5:** Main machining force comparison between the simulated and statistical values.

	Cutting Parameters			*F_main_* (N)	
Std Order	*V_c_* (m/min)	*F* (mm/rev)	*re* (mm)	FE Model	RegressionModel
1	80	0.08	0.40	182.3	176.3
2	80	0.11	0.40	194.1	190.6
3	80	0.14	0.40	213.6	212.9
4	115	0.08	0.40	186.5	182.8
5	115	0.11	0.40	201.0	200.3
6	115	0.14	0.40	221.3	225.8
7	150	0.08	0.40	190.2	190.5
8	150	0.11	0.40	206.5	211.1
9	150	0.14	0.40	233.7	239.8
10	80	0.08	0.80	207.7	236.0
11	80	0.11	0.80	250.2	256.2
12	80	0.14	0.80	284.5	284.5
13	115	0.08	0.80	232.1	241.2
14	115	0.11	0.80	266.2	264.6
15	115	0.14	0.80	301.5	296.1
16	150	0.08	0.80	249.6	247.5
17	150	0.11	0.80	281.2	274.1
18	150	0.14	0.80	320.7	308.8
19	80	0.08	1.20	319.1	313.7
20	80	0.11	1.20	346.9	339.9
21	80	0.14	1.20	371.3	374.3
22	115	0.08	1.20	323.3	317.6
23	115	0.11	1.20	344.4	347.0
24	115	0.14	1.20	382.6	384.5
25	150	0.08	1.20	322.4	322.7
26	150	0.11	1.20	347.4	355.2
27	150	0.14	1.20	392.3	395.9

**Table 6:** Analysis of variance (ANOVA) results for the resultant machining force.

**Source**	**Degree of** **Freedom**	**Sum of Squares**	**Mean Square**	***f*-Value**	***p*-Value**
Regression	9	113,045	12,560.6	238.87	0.000
Residual Error	17	894	52.6		
Total	26	113,939			
R-sq (adj) = 98.80%					
**Term**	**PE** **Coefficient**	**SE** **Coefficient**	***t*-Value**	***p*-Value**	
Constant	158.1	59.8	2.64	0.017	
*V*	−0.109	0.611	−0.18	0.861	
*f*	−822	774	−1.06	0.303	
*re*	48.8	39.5	1.24	0.233	
*V* ^2^	0.00046	0.00242	0.19	0.853	
*f* ^2^	4504	3289	1.37	0.189	
*re* ^2^	56.6	18.5	3.06	0.007	
*V × f*	3.03	1.99	1.52	0.147	
*V × re*	−0.093	0.150	−0.62	0.540	
*f × re*	498	174	2.86	0.011	

**Table 7:** Confirmation of the prediction model for *F_main_.*

Test No.	Simulated *F_main_* (N)	Predicted *F_main_* (N)	Relative Error (%)
I	197.4	189.7	−3.9
II	242.1	257.0	6.2
III	316.4	341.3	7.9
IV	217.6	203.1	−6.6
V	248.8	275.6	10.8
VI	385.7	365.1	−5.3
